# Prevalence of Systemic Arterial Hypertension and Associated Factors Among Adults from the Semi-Arid Region of Pernambuco, Brazil

**DOI:** 10.5935/abc.20190145

**Published:** 2019-10

**Authors:** Emerson Rogério Costa Santiago, Alcides da Silva Diniz, Juliana Souza Oliveira, Vanessa Sá Leal, Maria Izabel Siqueira de Andrade, Pedro Israel Cabral de Lira

**Affiliations:** 1 Universidade Federal de Pernambuco - Programa de Pós-graduação em Nutrição, Recife, PE - Brazil; 2 Universidade Federal de Pernambuco - Núcleo de Nutrição, Vitória de Santo Antão, PE - Brazil

**Keywords:** Hypertension/prevention and control, Prevalence, Cardiovascular Diseases, Epidemiology, Blood Pressure, Risk Factors

## Abstract

**Background:**

Systemic arterial hypertension is a substantial public health problem responsible for millions of deaths per year worldwide. However, little is known about the epidemiology of this disease in areas distant from large urban centers in Brazil. Such information is necessary to plan health promotion strategies.

**Objective:**

To estimate the prevalence of hypertension and determine its associated factors in adults residing in the semi-arid region of the state of Pernambuco, Northeastern Brazil.

**Method:**

This is a cross-sectional study conducted with a random sample of male and female adults. Individuals with systolic blood pressure ≥ 140 mm/Hg and/or diastolic blood pressure ≥ 90 mm/Hg and those who reported being under treatment with antihypertensive drugs were considered hypertensive. We collected data on demographic, socioeconomic, behavioral, and anthropometric characteristics, as well as health and nutrition. The statistical analysis used Pearson’s chi-square test, the chi-square test for trend, and multivariate Poisson regression analysis. A p-value < 0.05 in the final model was considered indicative of statistical significance.

**Results:**

The sample consisted of 416 individuals, and the prevalence of hypertension was 27.4% (95%CI 23.2 - 32.0). In the final model, the independent predictors of hypertension were age of 40 years or older (p = 0.000), low economic class (p = 0.007), smoking (p = 0.023), overweight determined by the body mass index (p = 0.003), and reduced glucose tolerance/diabetes mellitus (p = 0.012).

**Conclusion:**

The prevalence of hypertension was high and related to important risk factors. Thus, prevention and control strategies are recommended.

## Introduction

Systemic arterial hypertension is a substantial public health problem around the world and the most common clinical condition found in primary care.^1,2^ This condition is responsible for approximately 9.4 million deaths per year worldwide.^3^ It is not only one of the major risk factors for other cardiovascular diseases,^2^ but also a syndrome with its manifestations, characteristics, and multifactor etiology.^4^

The prevalence of hypertension increased from approximately 25.9% of the global adult population at the beginning of the 21^st^ century to 31.1% in 2010, a 5.2% increase in ten years.^5^ In developed countries, however, a 2.6% reduction occurred in this period, whereas developing countries faced a 7.7% increase.^5^ In Brazil, studies compiling data from several cities report that hypertension affects approximately 30% of the adult population, corresponding to 36 million individuals.^4,6,7^

Analyzing the distribution of the disease in the country, the north and northeast regions have the lowest rates of hypertension.^8^ However, this type of information is scarce in these regions due to the low number of surveys addressing the epidemiology of this condition.^9^

The semi-arid region covers a large area of Brazil, especially in the northeast part of the country. This region is often hit by crises related to long periods of drought. Besides the low socioeconomic development of the region, this situation can contribute to an increase in chronic non-communicable diseases.^10,11^ Nevertheless, little is known about the epidemiology of hypertension and its geographic distribution in populations living distant from large Brazilian urban centers and in mesoregions, such as the semi-arid region.

Considering the need for information that can assist in improving and optimizing public health services and actions, the present study aimed to estimate the prevalence of hypertension and determine its associated factors in the adult population from the semi-arid region of the state of Pernambuco, Northeastern Brazil.

## Methods

This is a population-based cross-sectional study conducted with male and female adults (aged 20 to 59 years) residing in the semi-arid region of Pernambuco.

The study population was determined by cluster sampling. Pernambuco is subdivided into 12 development regions (DR), six of which correspond to the semi-arid zone. Among them, three were randomly selected in the first stage of the sampling process. Next, one city was chosen from each DR: Serra Talhada (DR 4), Custódia (DR 12), and Belém de São Francisco (DR 1). Next, five census tracts were drawn per city with urban/rural distribution based on data from the 2010 Census. Lastly, 350 households were randomly selected to form a representative sample of the population in the semi-arid region of Pernambuco. The sample comprised all adult residents of the selected homes who were present at the time of data collection. Individuals with any physical limitation that hindered the anthropometric evaluation, debilitating diseases, and who declined to participate were excluded from the study. All stages of the selection process were performed using lists of random numbers generated with the aid of the EPITABLE tool of the Epi Info statistical package, version 6.04 (CDC/WHO, Atlanta, GE, USA).

The fieldwork occurred between July and September 2015 by a team of researchers who had previously undergone training for the administration of data collection instruments. A pilot study was conducted with 30 families in a city not selected for the main study to put into practice the logistics of the fieldwork and test the data collection instruments.

The sample size was calculated *a posteriori* assuming a 20% estimated prevalence of hypertension in the northeast region of Brazil,^8^ a 5% sampling error, a 95% confidence interval, and a factor of 1.5 to compensate for the design effect of the cluster sampling. Moreover, 10% was added to compensate for possible dropouts, leading to a total of 410 individuals.

The following demographic and socioeconomic characteristics and respective categories were collected: gender (male or female), age in years (20 to 29, 30 to 39, 40 to 49, 50 to 59), ethnicity (white or multiracial/black), schooling (never studied, primary school, high school/university), employment status (works or does not work), and place of residence (urban or rural area). Data collection followed the guidelines of the *Instituto Brasileiro de Geografia e Estatística* (IBGE - Brazilian Institute of Geography and Statistics).^12^ Economic class was categorized based on the Brazilian Economic Classification Criteria of the *Associação Brasileira de Empresas de Pesquisa* (ABEP - Brazilian Market Research Association):^13^ upper/middle (A1, A2, B1, B2, C1, C2) and lower (D, E) classes.

We collected the following behavioral characteristics: alcohol intake in the previous 30 days (yes or no); active smoking (smoker/ex-smoker or never smoked); passive smoking (yes or no; individuals who do not actively smoke but are frequently in contact with cigarette smoke from people at home, work, or school/university); and addition of salt to food after preparation (never, sometimes/almost always). Physical activity level was determined using the International Physical Activity Questionnaire (IPAQ) validated for use in Brazil,^14^ which enables the classification of individuals as sedentary/insufficiently active or active/very active.^14^

Anthropometric data were collected in duplicate following the guidelines of the World Health Organization.^15^ Body mass was measured using a digital scale (TANITA™, model BF-683 W). Height was measured using a portable stadiometer (Alturaexata™). Waist circumference (WC) was measured at the midpoint between the last rib and the iliac crest with a flexible, non-elastic metric tape (Sanny™). When a difference greater than 0.5 cm was found between the two height and WC values, the participant was measured a third time, and the two closest results were considered to calculate the arithmetic mean.

The following health and nutritional characteristics and respective categories were collected: body mass index (BMI) (not overweight when < 25 kg/m² and overweight when ≥ 25 kg/m²);^16^ WC (normal when < 80 for women and < 94 cm for men and increased when ≥ 80 cm for women and ≥ 94 cm for men);^16^ waist-to-height ratio (normal when < 0.52 for men and < 0.53 for women and increased when ≥ 0.52 for men and ≥ 0.53 for women);^17^ and food security evaluated using the Brazilian Food and Nutritional Insecurity Scale,^18^ which enabled classifying the homes into the following categories: food security, mild food insecurity and moderate/severe food insecurity.

Blood samples were collected through a venous puncture after a 10-hour fast. The analyses to determine the levels of fasting blood glucose, triglycerides, and total cholesterol used the Accutrend GCT [Roche Diagnóstica, Brazil], which allows immediate readings. The components of the biochemical profile were fasting blood glucose [normal when < 100 mg/dL and reduced glucose tolerance/diabetes mellitus (DM) when ≥ 100 mg/dL or when the individual used a hypoglycemic medication],^19^ triglycerides (normal when < 150 mg/dL and high when ≥ 150 mg/dL),^20^ and total cholesterol (normal when < 190 mg/dL and high when ≥ 190 mg/dL).^20^

Regarding the outcome variable, blood pressure (BP) was measured in duplicate using the auscultation method (Glicomed™ sphygmomanometer, model CE-0483), followed by the calculation of the arithmetic mean of the results. The procedures to prepare the individuals for BP measurement followed the recommendations of the Brazilian Society of Cardiology:^6^ make sure that the individual rested for at least five minutes in a calm environment; did not have a full bladder, had not practiced physical exercise in the previous 60 minutes, had not consumed alcohol, coffee, or food in the previous hour, and had not smoked in the previous 30 minutes; and was seated at the time of the measurement, with the legs uncrossed, feet flat on the floor, and arm at the height of the heart. The criteria to diagnose hypertension was based on the Seventh Brazilian Hypertension Guidelines,^6^ which classify an individual with hypertension when the systolic BP is ≥ 140 and/or diastolic BP is ≥ 90. We also considered hypertensive individuals who declared having a previous diagnosis and were under treatment with antihypertensive medications.

The data used in the present investigation derived from a study entitled “Evaluation of food and nutritional security in urban and rural communities affected by drought in the semi-arid region of Pernambuco” (certificate of presentation for ethical approval: 38878814.9.0000.5208; certificate of approval: 897.655). All participants received information about the study and signed the informed consent form.

### Statistical analysis

All data were entered twice with the Epi Info™ software, version 6.04 (CDC/WHO, Atlanta, GE, USA), with the subsequent use of the VALIDATE module to check data consistency. We grouped the explanatory variables into the following four hierarchically ordered levels from distal to proximal: 1) biological factors; 2) demographic and socioeconomic factors; 3) behavioral factors, and 4) biochemical and nutritional factors (proximal level). Based on a conceptual model to determine hypertension, we assumed that predisposing factors imply different hierarchical levels of determination.

We conducted univariate statistical analysis with either Pearson’s chi-square test or the chi-square test for trend to establish associations between explanatory variables and the outcome. Variables with a p-value < 0.20 were incorporated into the multivariate analysis using Poisson regression with robust variance. Results of the univariate analysis were expressed as percentages and respective 95% confidence intervals (95%CI) and of the multivariate analysis were described as prevalence ratios and respective 95%CI. A p-value < 0.05 in the final model was considered indicative of a statistically significant association. All analyses had the aid of the Statistical Package for Social Sciences (SPSS), version 13.0 (IBM Analytics, NC, USA) and Stata, version 14.0 (StataCorp, TX, USA).

## Results

The final sample consisted of 416 adults with a median age of 35.0 (interquartile range of 28.0 to 48.0) years. Most of the sample was female (64;9%, 95%CI: 60.1 to 69.5), of black/multiracial ethnicity (78.4%, 95%CI: 74.0 to 82.2), and lived in urban areas (57.9%, 95%CI: 53.0 to 62.7).

A total of 19.7% (95%CI: 16.1 to 23.9) of the sample had the habit of consuming alcoholic beverages, 23.3% (95%CI: 19.4 to 27.7) smoked actively, 16.3% (95%CI: 13.0 to 20.3) smoked passively, and 71.5% (95%CI: 65.6 to 76.9) were sedentary or insufficiently active. Moreover, 10.1% (95%CI: 7.5 to 13.5) of the sample reported adding salt to food after preparation sometimes or nearly always.

The prevalence of hypertension was 27.4% (95%CI: 23.2 to 32.0). Table 1 shows the distribution of the condition according to demographic and socioeconomic variables. We found a statistically significant association between higher prevalence of hypertension and increasing age and lower levels of schooling and income. Regarding behavioral variables (Table 2), hypertension was more frequent among active smokers/ex-smokers and passive smokers. With respect to the health and nutritional profile (Table 3), hypertension was associated with overweight, determined by the BMI, and an increased weight-to-height ratio. Hypertension was also associated with the following biochemical variables: reduced glucose tolerance/DM and high total cholesterol (Table 4).

**Table 1:** Distribution of systemic arterial hypertension according to demographic and socioeconomic characteristics in adults from the semi-arid region, state of Pernambuco, Brazil, 2015

	Systemic arterial hypertension			
Variables	n	%	95%CI	p-value‡
**Gender**				**0.358**
Male	44	30.1	22.8 – 38.3	
Female	70	25.9	20.8 – 31.6	
**Age (years)**				**0.000§**
20 – 29	13	10.6	05.7 – 17.4	
30 – 39	20	15.9	10.0 – 23.4	
40 – 49	25	36.8	25.4 – 49.3	
50 – 59	56	56.6	46.2 – 66.5	
**Ethnicity**				**0.721**
White	26	28.9	19.8 – 39.4	
Multiracial or black	88	27.0	22.3 – 32.2	
**Schooling**				**0.000§**
Never studied	66	44.6	36.4 – 53.0	
Primary school	32	19.5	13.7 – 26.4	
High school/university	16	15.4	09.1 – 23.8	
**Employment status**				**0.150**
Works	45	23.9	18.0 – 30.7	
Does not work	69	30.3	24.4 – 36.7	
**Place of residence**				
Urban area	65	27.0	21.5 – 33.0	0.816
Rural area	49	28.0	21.5 – 35.3	
**Economic class**				**0.001**
Upper or middle*	45	20.5	15.3 – 26.4	
Lower†	69	35.2	28.5 – 42.3	

95%CI: 95% confidence interval;

* classes A1, A2, B1, B2, C1, and C2;

† classes D and E;

‡ Pearson’s chi-square test;

§ chi-square test for trend.

**Table 2:** Distribution of systemic arterial hypertension according to behavioral characteristics in adults from the semi-arid region, state of Pernambuco, Brazil, 2015

Variables	Systemic arterial hypertension			p-value§
	n	%	95%CI	
Alcohol intake*				**0.330**
Yes	26	31.7	21.9 – 42.9	
No	88	26.3	21.8 – 31.5	
**Active smoking**				**0.000**
Smoker or ex-smoker	54	55.7	45.2 – 65.8	
Never smoked	60	18.8	14.8 – 23.6	
**Passive smoking†**				**0.000**
Yes	32	47.1	34.8 – 59.6	
No	82	23.6	19.3 – 28.4	
**Physical activity level‡**				**0.078**
Sedentary or insufficiently active	61	32.4	25.8 – 39.6	
Active or very active	33	44.0	32.5 – 55.9	
**Addition of salt to food**				**0.200**
Never	106	28.3	23.9 – 33.3	
Sometimes or almost always	8	19.0	08.6 – 34.1	

95%CI: 95% confidence interval;

* considering the 30 days prior to data collection;

† individuals who do not actively smoke but are frequently in contact with cigarette smoke from people at home, work, or school/university;

‡ classified using the International Physical Activity Questionnaire (IPAQ);

§ Pearson’s chi-square test.

**Table 3:** Distribution of systemic arterial hypertension according to health and nutritional characteristics in adults from the semi-arid region, state of Pernambuco, Brazil, 2015

Variables	Systemic arterial hypertension			p-value**
	n	%	95%CI	
BMI				0.019
Not overweight*	29	19.9	13.7 – 27.3	
Overweight†	76	30.6	25.0 – 36.8	
WC				0.082
Normal‡	29	21.8	15.1 – 29.8	
Increased§	81	30.0	24.6 – 35.8	
Waist-to-height ratio				0.012
Normal//	17	17.0	10.2 – 25.8	
Increased¶	87	29.8	24.6 – 35.4	
Food security#				0.245††
Food security	35	34.0	24.9 – 44.0	
Mild food insecurity	33	23.7	16.9 – 31.7	
Moderate or severe food insecurity	46	26.4	20.1 – 33.6	

95%CI: 95% confidence interval; BMI: body mass index; WC: waist circumference;

* BMI < 25.0 kg/m^2^;

† BMI ≥ 25.0 kg/m^2^;

‡ < 80 cm for women and < 94 cm for men;

§ ≥ 80 cm for women and ≥ 94 cm for men;

// < 0.52 for men and < 0.53 for women;

¶ ≥ 0.52 for men and ≥ 0.53 for women;

# classified according to the Brazilian Food and Nutritional Insecurity Scale;

** Pearson’s chi-square test;

†† chi-square test for trend.

**Table 4:** Distribution of systemic arterial hypertension according to biochemical variables in adults from the semi-arid region, state of Pernambuco, Brazil, 2015

Variables	Systemic arterial hypertension			p-value#
	n	%	95%CI	
**Fasting blood glucose**				**0.000**
Normal*	56	28.4	22.2 – 35.3	
Reduced glucose tolerance and/or DM†	30	68.2	52.4 – 81.4	
**Triglycerides**				**0.416**
Normal‡	32	32.7	23.5 – 42.9	
High§	54	37.8	29.8 – 46.2	
**Total cholesterol**				**0.005**
Normal//	27	25.7	17.7 – 35.2	
High¶	59	43.4	34.9 – 52.1	

95%CI: 95% confidence interval; DM: diabetes mellitus;

* < 100 mg/dL;

† ≥ 100 mg/dL or when hypoglycemic medication was used;

‡ < 150 mg/dL;

§ ≥ 150 mg/dL;

// < 190 mg/dL;

¶ ≥ 190 mg/dL;

# Pearson’s chi-square test.

After statistical adjustments in the hierarchical model, the explanatory variables that remained significantly associated with hypertension were age, economic class, active smoking, BMI, and fasting blood glucose (Table 5).

**Table 5:** Crude and adjusted prevalence ratios for systemic arterial hypertension according to explanatory variables in adults from the semi-arid region, state of Pernambuco, Brazil, 2015

Variables	n	Systemic arterial hypertension				p-value§
		Crude analysis		Adjusted analysis		
		PR	95%CI	PR	95%CI	
**Age (years)**						
20 – 29	13	1.00		1.00		
30 – 39	20	1.05	0.97 – 1.13	1.05	0.97 – 1.13	0.214
40 – 49	25	1.24	1.12 – 1.36	1.24	1.12 – 1.36	0.000
50 – 59	56	1.42	1.31 – 1.53	1.42	1.31 – 1.53	0.000
Economic class*						
Upper or middle	45	1.00		1.00		
Lower	69	1.12	1.05 – 1.20	1.09	1.02 – 1.17	0.007
**Active smoking†**						
Never smoked	54	1.00		1.00		
Smoker or ex-smoker	60	1.31	1.22 – 1.41	1.11	1.02 – 1.22	0.023
**BMI‡**						
Not overweight	29	1.00		1.00		
Overweight	76	1.09	1.02 – 1.17	1.21	1.07 – 1.37	0.003
**Fasting blood glucose‡**						
Normal	56	1.00		1.00		
Reduced glucose tolerance and/or DM	30	1.31	1.19 – 1.44	1.15	1.03 – 1.27	0.012

PR: prevalence ratio; 95%CI: 95% confidence interval; BMI: body mass index; DM: diabetes mellitus; PR 1.00 - reference;

* adjusted for age, schooling, and employment status;

† adjusted for age, schooling, employment status, economic class, passive smoking, and physical activity level;

‡ adjusted for age, schooling, employment status, economic class, passive smoking, physical activity level, BMI, WC, waist-to-height ratio, and fasting blood glucose;

§ Poisson regression with robust variance.

## Discussion

Hypertension is one of the most common conditions among older adults, but it also affects a considerable portion of the adult population (20 to 59 years), striking more than 30 million individuals in this age range in Brazil alone.^6^ Thus, addressing this condition in the adult population is necessary.

Although slightly lower than the estimated national average of 30%,^6^ the prevalence of hypertension among adults in the semi-arid region of Pernambuco was high, confirming that this is a serious public health problem. This finding was expected, given the low socioeconomic development of the mesoregion and its possible association with the high prevalence of chronic non-communicable diseases.^10^ We underline, however, that some individuals classified as hypertensive may actually have “white coat hypertension,” which was not evaluated and could be considered a limitation of the present study.

According to Andrade et al.,^8^ the prevalence of self-reported hypertension among adults in Northeastern Brazil is 19.4% (95%CI: 18.4 to 20.5), which is lower than the rate found in the present study. This divergence may be explained by one of the limitations of using self-reports, which, although validated in population-based studies, might underestimate prevalence rates.^21^ This aspect is influenced by the access to and use of health care services by the part of the population investigated, as self-reported hypertension would require a previous medical diagnosis.^21^

The greater susceptibility to hypertension with the increase in age found in the present study has been reported in the specialized literature, and there is a consensus on the direct, linear relationship between BP and age.^6^ This relationship results from the development of atherosclerosis, with the stiffening of the arteries leading to an elevation in pressure levels, which is normally caused by physiological changes stemming from the aging process.^22^

The association between economic class and hypertension in the present study supports the conjecture that individuals with low status are more vulnerable to the development of the disease.^23^ Furthermore, despite the association with a low level of schooling having lost its significance in the multivariate model, it could represent a more evident risk factor than income.^23^ Thus, it is important to increase the monitoring of and care for these more vulnerable groups.

Being a smoker or ex-smoker was also associated with the prevalence of hypertension, which corroborates data from other population-based studies conducted in Brazil and a review study by Passos et al.^21^ This result is consistent with experimental evidence that smoking can cause hypertension and other cardiovascular diseases.^24^ In the first decade of the 21^st^ century, 11% of worldwide deaths from cardiovascular diseases were attributed to smoking,^25^ making this habit an important risk factor to address in health promotion and disease prevention actions.

Body composition is another important aspect related to hypertension, especially with regard to fat distribution, as the increase in visceral adipose tissue is directly associated with a greater incidence of the disease.^26^ One of the limitations of the present study was not evaluating body fat distribution based on more accurate methods, such as the quantification of visceral or subcutaneous adipose tissue using computed tomography.^27^ However, studies report that indicators such as BMI and WC are good tools to use in population-based studies and increases in these measures are associated with a higher risk of developing hypertension.^28-30^

The positive association between overweight based on BMI and hypertension in the semi-arid region of Pernambuco underscores the need for more effective dietary and nutritional education programs derived from health promotion policies and actions, in addition to greater encouragement to practice physical activity. Strategies of this nature would have a higher impact on the process of nutritional transition that has affected the country^31^ and culminated in a 26.3% increase in overweight between 2006 and 2016, according to a telephone survey conducted by the Brazilian Ministry of Health.^32^

The concomitant occurrence of hypertension and reduced glucose tolerance and/or DM supports the scientific evidence indicating the close link between these conditions, which often develop together and through the same metabolic pathways.^33^ An analysis of the Brazilian National Household Surveys conducted in 1998, 2003, and 2008 shows an increase from 1.7 to 2.8% in the prevalence coefficient standardized by gender and age range for DM associated with hypertension in the period, especially in the northeast and midwest regions of the country.^34^ These data further highlight the considerable problems these conditions represent, especially in regions such as Northeastern Brazil and mesoregions such as the semi-arid region.

The lack of associations between hypertension and alcohol intake, sedentary lifestyle, and the addition of salt to food after preparation was an unexpected finding, as these aspects are traditionally considered risk factors for the disease.^6^ This paradox may be attributed to reverse causality, which consists of the repercussion of a disease positively changing the behavior of individuals, as many respondents were aware of their hypertension at the time of data collection. Another possible explanation for the lack of such associations would be the sample size, which was calculated only to estimate the prevalence of the outcome, possibly limiting the robustness of sub-analyses. Also, the predominance of females in the present study may have been due to the sampling method adopted.

The cross-sectional design constitutes another limitation of the present study by not allowing the inference of causality, as information on exposure and outcome are collected at the same time. However, this study makes important contributions to the knowledge about the epidemiology of hypertension in the population investigated. According to Vianna and Segall-Corrêa,^35^ initiatives such as the present study are important and necessary for the acquisition of previously unpublished information that can be used for regional, national, and international comparisons. Cross-sectional studies conducted in specific locations can provide better knowledge of local aspects, peculiarities, and risk factors that could go unnoticed in analyses involving broader territorial units, thereby complementing information on the geographic distribution of the disease.

## Conclusion

The prevalence of hypertension was high in the semi-arid region studied and was associated with important risk factors, such as increasing age, low socioeconomic class, active smoking, overweight, and reduced glucose tolerance and/or DM. The constant monitoring of chronic non-communicable diseases, especially hypertension, DM, and obesity, and their associated factors is fundamental to the planning and continuous improvement of public health programs and actions, as well as the drafting of specific strategies for the region studied.
